# Expression of MMP-2, MMP-9, and NGAL in Tissue and Serum of Patients with Vascular Aneurysms and Their Modulation by Statin Treatment: A Pilot Study

**DOI:** 10.3390/biom10030359

**Published:** 2020-02-26

**Authors:** Erika Cione, Elena Piegari, Giuseppe Gallelli, Maria Cristina Caroleo, Elena Lamirata, Francesca Curcio, Federica Colosimo, Roberto Cannataro, Nicola Ielapi, Manuela Colosimo, Stefano de Franciscis, Luca Gallelli

**Affiliations:** 1 Department of Pharmacy, Health and Nutritional Sciences, Department of Excellence 2018-2022, University of Calabria, 87036 Rende, Italy; erika.cione@unical.it (E.C.); mariacristinacaroleo@virgilio.it (M.C.C.); r.cannataro@gmail.com (R.C.); 2 Department of Experimental Medicine, Section of Pharmacology, University of Campania “Luigi Vanvitelli”, 80138 Napoli, Italy; elena.piegari@unicampania.it; 3 Unit of Vascular Surgery, Department of Surgery, “Pugliese Ciaccio” Hospital, 88100 Catanzaro, Italy; giuseppegallelli@hotmail.it; 4 Department of Experimental Medicine, University of Catanzaro, and Vascular Surgery Unit, 88100 Mater Domini Hospital, 88100 Catanzaro, Italy; elena.lamirata@libero.it (E.L.); francesca.curcio@icloud.com (F.C.); defranci@unicz.it (S.d.F.); 5 National Institution of Social Insurance, Department of Medical Law, 88100 Catanzaro, Italy; federicacolosimo@virgilio.it; 6 Department of Public Health and Infectious Disease, “Sapienza” University of Rome 5, 00185 Roma, Italy; infermierenicola@hotmail.it; 7 Unit of Microbiology and Virology, “Pugliese Ciaccio” Hospital, 88100 Catanzaro, Italy; manuelacolosimo@hotmail.it; 8 Department of Health Sciences, University of Catanzaro, and Clinical Pharmacology and Pharmacovigilance Unit, Mater Domini Hospital, 88100 Catanzaro, Italy

**Keywords:** MMPs, NGAL, statins, arterial aneurysms, patients

## Abstract

Background: Matrix metalloproteinases (MMPs) are involved in vascular wall degradation, and drugs able to modulate MMP activity can be used to prevent or treat aneurysmal disease. In this study, we evaluated the effects of statins on MMP-2, MMP-9, and neutrophil gelatinase-associated lipocalin (NGAL) in both plasma and tissue in patients with aneurysmal disease. Methods: We performed a prospective, single-blind, multicenter, control group clinical drug trial on 184 patients of both sexes >18 years old with a diagnosis of arterial aneurysmal disease. Enrolled patients were divided into two groups: Group I under statin treatment and Group II not taking statins. In addition, 122 patients without aneurysmal disease and under statin treatment were enrolled as a control group (Group III). The expression of MMPs and NGAL in plasma was evaluated using ELISA, while their expression in endothelial tissues was evaluated using Western blot. Results: The ELISA test revealed greater plasma levels (*p* < 0.01) of MMPs and NGAL in Groups I and II vs. Group III. Western blot analysis showed higher expression (*p* < 0.01) of MMPs and NGAL in Group II vs. Group I, and this increase was significantly higher (*p* < 0.01) in patients treated with low potency statins compared to high potency ones. Conclusions: MMPs and NGAL seem to play a major role in the development of aneurysms, and their modulation by statins suggests that these drugs could be used to prevent arterial aneurysmal disease.

## 1. Introduction

An aneurysm is the dilatation of the aorta induced by vascular wall degradation that can localize centrally or peripherally, affecting all portions of the aortic tube [1]. Several biochemical mediators are involved in the vascular wall degradation of the aorta, e.g., neutrophil elastase, serine proteases, and matrix metalloproteinases (MMPs) [2]. This latter class of enzymes is divided into collagenases (MMP-1, MMP-8, MMP-13, MMP-18), stromolysins (MMP-3 and MMP-10), matrilysins (MMP-7 and MMP-26), and gelatinases (MMP-2 and MMP-9), according to their protein degradation capabilities. In general, MMPs are able to degrade most components of the extracellular matrix (ECM) of the vessel wall, leading to an imbalance between ECM synthesis and degradation [3]. MMP-2 and MMP-9 are the most common MMPs involved in endothelial degradation [4,5,6]. Their activity chiefly depends on the balance between the formation of complexes containing MMP-9, neutrophil gelatinase-associated lipocalin (NGAL), and the expression of MMP tissue inhibitors (TIMPs) [7]. An imbalance between these factors results in uncontrolled changes in the ECM, which may lead to a widening of the aneurysm vessel with a future aortic aneurysm rupture [3,8,9,10]. Previously, we reported that plasma MMP-9 and NGAL levels were significantly elevated in patients with cerebral artery aneurysms [11]. Moreover, we also documented that several drugs/compounds can modulate the activity of MMPs, improving a patient’s clinical outcomes [12,13]. It has been documented that HMG-CoA reductase inhibitors, also known as statins, inhibit the synthesis of cholesterol in the liver and can decrease arterial aneurysm growth rates by stabilizing endothelial function [14,15,16] and reducing inflammation and oxidative stress [17,18]. In this pilot clinical trial, we evaluated the effects of statins on MMP-2, MMP-9, and NGAL plasma levels in patients with aneurysmal disease.

## 2. Materials and Methods

### 2.1. Study Design

We performed a prospective, single-blind, multicenter, control group study from October 2017 to October 2019 in subjects admitted to the Department of Medical and Surgical Science at University Magna Graecia, Catanzaro and to the Unit of Vascular and Endovascular Surgery at the Regional Hospital “Pugliese Ciaccio”, Catanzaro.

The study was approved by the Investigational Review Board, in accordance with the Declaration of Helsinki and the Guideline for Good Clinical Practice. Before the beginning of the study, all participants provided written informed consent.

### 2.2. Patients

In this prospective, pilot clinical drug trial, we enrolled patients of both sexes >18 years old with aneurysmal disease that had been diagnosed clinically and instrumentally. Patients who had not signed the informed consent form were excluded from the study. The diagnosis of aneurysmal disease was determined by assessing patient presentation and vascular examination, including an ankle–brachial index of <0.9 (in peripheral diseases) and duplex ultrasonography.

### 2.3. Experimental Study

Enrolled patients were divided into two groups: Group I (statin-treated): patients with a central or peripheral aneurysm under statin treatment; Group II (statin-free): patients with a central or peripheral aneurysm without a history of statin use. The cardiovascular risk factors that were analyzed included a history of smoking, diabetes mellitus, hypertension, hyperlipidemia, and coronary artery disease based on a presenting thallium nuclear stress test or transthoracic echocardiogram. Chronic renal failure was defined as serum creatinine level >1.5 mg/dL. 

At the time of admission, each patient underwent a baseline contrast-enhanced computed tomography scan for diagnosis of the disease and for evaluation of the aneurysm length, maximum aneurysm diameter, diameter of the access vessels, and presence or absence of a wall thrombus at the level of the central or peripheral aneurysm. Follow-ups were performed at 1 (T1) and 3 (T2) months after surgery. Finally, patients >18 years old without aneurysmal disease, but treated with statins, were enrolled as **Group III** and were defined as a control group.

### 2.4. Endpoints

*Primary endpoint:* Statistically significant difference (*p* < 0.05) in the plasma levels of MMP-2, MMP-9, and NGAL between patients with central and peripheral aneurysms treated or not treated with HMG-CoA reductase inhibitors. In addition, the statistically significant difference (*p* < 0.05) of plasma levels of MMP-2, MMP-9, and NGAL among patients with aneurysmal disease taking statins at high and low doses/potencies. 

*Secondary endpoint:* Correlation between the expression of MMP-2, MMP-9, and NGAL in plasma and tissue levels and the maximum diameter of the aneurysm. 

*Safety endpoint:* Development of muscle pain in patients under statin treatment compared to the patients free of statins and correlation with plasma levels of MMP-2, MMP-9, and NGAL.

### 2.5. Enzyme-Linked Immunosorbent Assay (ELISA)

In patients enrolled in Groups I and II, to evaluate the plasma levels of MMP-2, MMP-9, and NGAL, blood samples collected at the time of admission (T0) and during the follow-ups (T1 and T2) were frozen at −80 °C for ELISA evaluation. In contrast, in patients enrolled in Group III, blood collected during the routine evaluation for lipid plasma levels (T0) was frozen at −80 °C for ELISA evaluation. The ELISA test was conducted according to our previous study [19,20,21]. Commercially available sandwich ELISA kits for the inactive form of MMP-2, MMP-9, and NGAL (Biotrak MMP-2 and MMP-9 Human ELISA System, Amersham Pharmacia Biotech, Buckinghamshire, UK; NGAL Human ELISA System, Bioporto Diagnostics, Gentofte, Denmark) were used, following the manufacturers’ instructions. The absorbance of each well was measured with a microtiter plate reader (Synergy H1, BioTeck, Winooski, VT, USA).

### 2.6. Protein Extraction and Immunoblot Analysis

Western blot analysis was performed in agreement with our previous studies [22,23,24] and only on tissue derived from arterial aneurysms (Groups I and II). About 40 mg of arterial aneurysm obtained by removing the aneurysm wall during the surgical procedures was immediately washed in ice-cold physiological saline. In agreement with the instructions of the total protein extraction kit, the tissues were lysed in 2 mL of tissue protein extraction reagent (25 mmol/L bicine, 150 mmol/L sodium chloride pH 7.6 (Thermo Scientific, Waltham, MA, USA)) and then were placed in ice for homogenization by grinding. Tissue homogenates were centrifuged twice for 10 min at 12,000× *g* and 4 °C. Supernatants were collected and proteins were quantified according to the instructions of the Quant-iT™ Protein Assaykit (Thermo Fisher). They were then lyophilized and resuspended in 100 µL tissue protein extraction reagent, and an equal amount of protein extracts was then separated using 15% sodium dodecyl sulfate–polyacrylamide gel electrophoresis (SDS-PAGE) under constant voltage (about 150 V) and transferred to nitrocellulose membranes (Amersham Pharmacia, Little Chalfont, UK). Immunoblotting of protein was performed using anti-MMP-2, anti-MMP-9, and anti-NGAL (abcam #ab86607, #ab119906, and #ab23477, respectively) monoclonal primary antibodies (1:1000); each one was separately incubated for 1 h at room temperature in TBST 1x and 5% of milk under shake. Then, goat antimouse IgG-HRP (abcam #ab205719) secondary antibody (1:5000) was incubated for 2 h at room temperature in TBST 1x as described [7]. Loading conditions were determined with polyclonal GAPDH (Sigma-Aldrich) primary antibody (1:1000) after membrane stripping for 1 h at room temperature in TBST 1x and 5% of milk under shake. Then, goat antirabbit IgG-HRP (Santa Cruz #sc-2004) secondary antibody (1:10,000) was incubated for 2 h at room temperature in TBST 1x. Bands were visualized by enhanced chemiluminescence (ECL Plus, Amersham Pharmacia); the intensities of experimental bands were analyzed and normalized by loading control GAPDH by computer-assisted densitometry using Photoshop and then expressed as arbitrary units. All experiments were performed in triplicate.

### 2.7. Safety Evaluation

The development of adverse drug reactions (ADRs) (e.g., liver toxicity and myalgia) and drug–drug interactions were evaluated using the Naranjo scale and the drug interactions probability scales, respectively, in agreement with our previous studies [25,26,27,28].

### 2.8. Statistical Analysis

**Figure 1:**
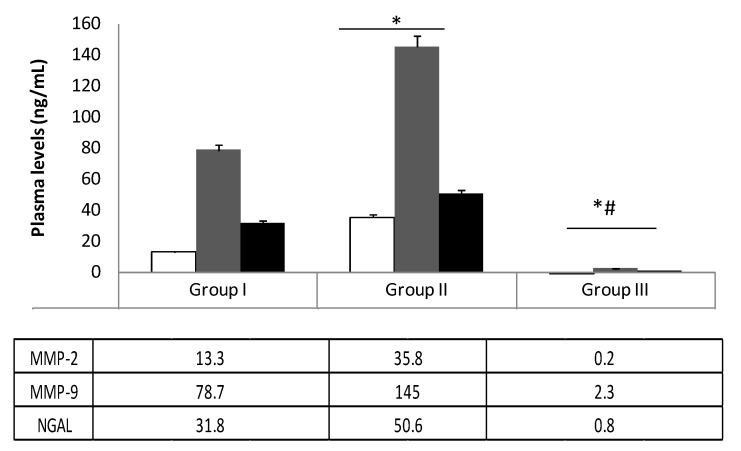
Plasma levels of matrix metalloproteinase (MMP)-2, MMP-9, and neutrophil gelatinase-associated lipocalin (NGAL) in enrolled patients at the time of admission (T = 0). Group I: aneurysmal patients using statins; Group II: aneurysmal patients, statin-free; Group III: control patients. Group III vs. Group I, * *p* < 0.01; Group III vs. Group II, # *p* < 0.01.

**Figure 2:**
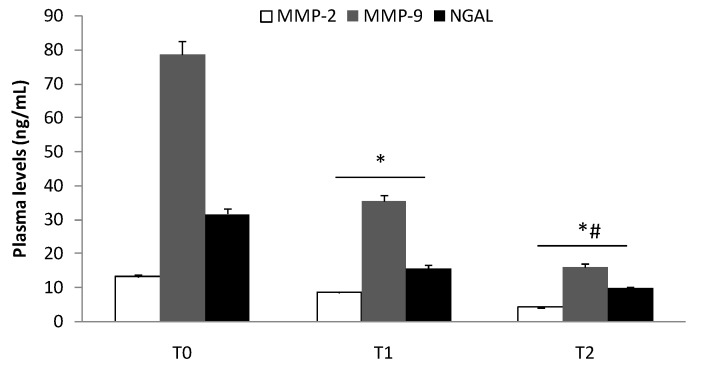
Plasma levels of MMP-2, MMP-9, and NGAL in aneurysmal patients under statin treatment (Group I) at the time of admission (T0) and 1 (T1) and 3 (T2) months later. T1 vs. T0, * *p* < 0.01; T2 vs. T1, # *p* < 0.01.

**Figure 3:**
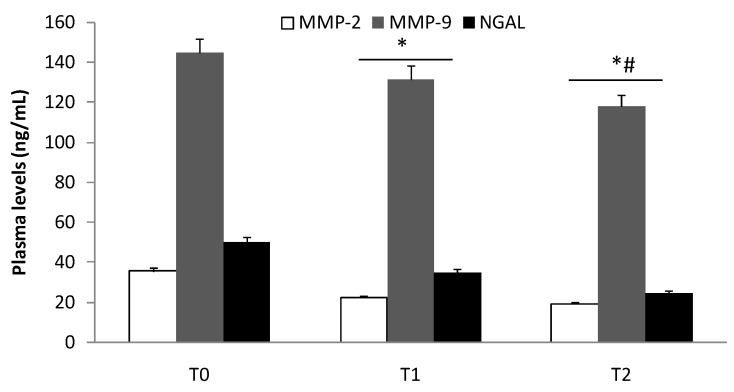
Plasma levels of MMP-2, MMP-9, and NGAL in aneurysmal patients free of statin treatment (Group II) at the time of admission (T0) and 1 (T1) and 3 (T2) months later. T1 vs. T0, * *p* < 0.05; T2 vs. T1, # *p* < 0.05.

**Figure 4:**
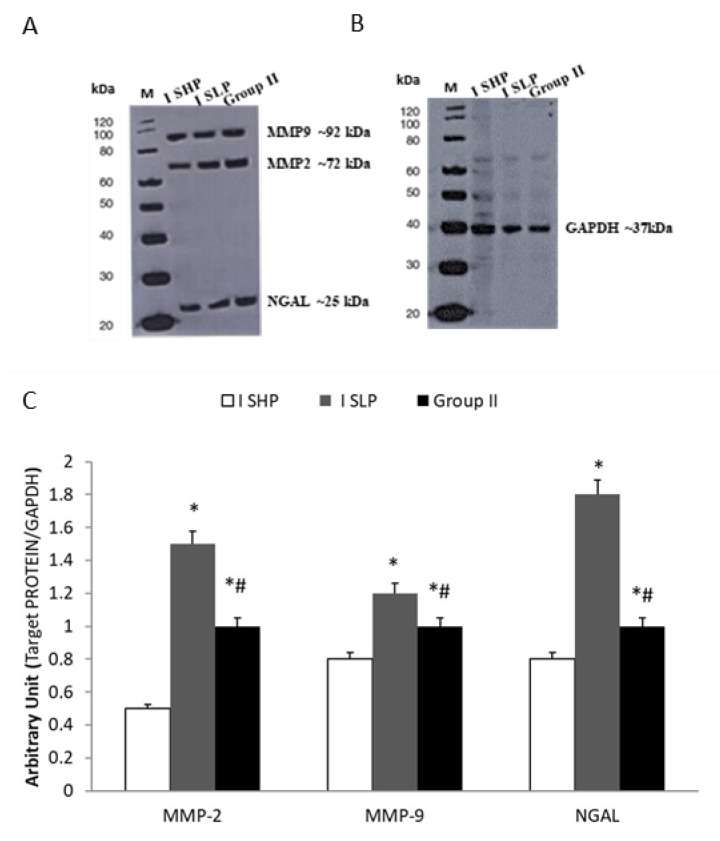
(**A**) Representative tissue levels of MMP-2, MMP-9, and NGAL in aneurysmal tissue. (**B**) GAPDH is used as loading control. (**C**) Tissue expression of MMP2, MMP-9, and NGAL in patients enrolled in Group I and treated with statin high potency (I SHP) (taking rosuvastatin 40 mg/day or atorvastatin 80 mg/day) or statin low potency (I SLP) (taking rosuvastatin <40 mg/day or atorvastatin <80 mg/day) and in patients enrolled in Group II (statin-free treatment). The results are expressed as mean ± standard error of the mean (SEM); * *p* < 0.01 vs. Group I SHP; # *p* < 0.01 vs. Group I SLP.

Patient characteristics data (Table 1) are expressed as absolute values or as mean ± standard deviation. MMP and NGAL expression data (Figure 1, Figure 2, Figure 3 and Figure 4) are represented as mean values ± standard error of the mean (SEM). The Student’s t-test was performed to analyze the difference between each group and the control. Analysis of variance was used to evaluate the difference between the groups. The threshold of statistical significance was set at * *p* < 0.05. SPSS (SPSS Inc., Chicago, IL, USA) software was used for statistical analyses. We defined and labeled this study as exploratory; therefore, we did not determine a power calculation. 

## 3. Results

### 3.1. Patients

In this study, we enrolled 184 patients—123 males (mean age 73.2 ± 8.6 years) and 61 females (mean age 68.7 ± 4.1 years)—with a diagnosis of central or peripheral aneurysmal disease: 15 in the popliteal artery (APA = 8.1%), 6 in the femoral artery (AFA = 3.3%), 28 in the iliac artery (AIA = 15.2%), and 135 in the abdominal aorta (AAA = 73.4%). Group I statin-treated: there were 65 patients—45 males (mean age 73.5 ± 8.4 years) and 20 females (mean age 69.4 ± 4.3 years)—taking rosuvastatin 10–40 mg/day or atorvastatin 20–80 mg/day. In this group, we designated further subgroups as statin high potency (SHP) (taking rosuvastatin 40 mg/day or atorvastatin 80 mg/day) and statin low potency (SLP) (taking rosuvastatin <40 mg/day or atorvastatin <80 mg/day). Group II statin-free: there were 119 patients—78 males (mean age 72.9 ± 8.8 years) and 41 females (mean age 68.3 ± 4.0 years)—who had never used statins as pharmacological treatment. Both groups had true aneurysms and had not received a vascular intervention or any other operative vascular interventions within the 3 years prior to inclusion in this study. 

The control group was classified as Group III. In this group, there were 122 patients—68 males (median age 71 ± 7.3) and 54 females (median age 67.5 ± 5.2)—under statin treatment; they were screened with duplex ultrasonography to exclude the presence of asymptomatic central or peripheral aneurysms. Finally, in 58 patients of Group I (61%) and in 65 patients of Group III (53.3%), adverse drug reactions related to statin use were present. Despite this, statin treatment was not dismissed. 

Patients’ characteristics are shown in Table 1.

### 3.2. Elisa Test of MMP-2, MMP-9, and NGAL Plasma Levels

ELISA biochemical determination revealed greater plasma levels (*p* < 0.01) of MMP-2 and MMP-9 in patients with aneurysms (Groups I and II), compared to control patients (Group III); lower levels of MMP-2, MMP-9, and NGAL (*p* < 0.01) were found in patients treated with statins (Group I) compared with untreated patients (Group II) (Figure 1).

The same trend was evident in Groups I and II (Figure 2 and Figure 3; *p* < 0.05). The reduction (T2) remained significantly higher (*p* < 0.01) compared to patients enrolled in Group III (Figure 4). Finally, we did not record any difference in the expression of MMP-2 and MMP-9 in patients who developed myalgia during statin treatment.

### 3.3. Protein Expression Levels of MMP-2, MMP-9, and NGAL in Aneurysmal Tissues

Western blot analysis showed increased protein expression (*p* < 0.01) of MMP-2, MMP-9, and NGAL in tissues of statin-untreated patients (Group II) compared to statin-treated patients (Group I). This increase was significantly higher (*p* < 0.01) in patients receiving low potency statins (Group I SLP) compared to patients treated with high potency statins (Group I SHP) (Figure 4A–C).

## 4. Discussion

In this study, we evaluated the expression of MMP-2, MMP-9, and NGAL in the plasma and tissue of patients with aneurysmal diseases. The modulation of these three biochemical indicators of aortic integrity in patients under statin treatment was also studied. Recently, in a preclinical experimental model, Krueger et al. [29] documented that vascular damage is associated with the upregulation of MMPs, which are involved in elastin degradation. An uncreated expression of MMPs causes loss of elastin and can induce compensatory fibrosis and inflammation with the destruction of all major matrix components, excessive distension, and vessel wall rupture [30,31,32]. In this view, recently, we documented that vascular damage induces an inflammatory response that can evoke a local reaction with the activation and recall of cytokines and inflammatory mediators [33] involved in aneurysmal diseases [32,34]. In an interesting pilot study, Yuwen et al. [35], investigating the expression of a plethora of cytokines in patients with aortic aneurysmal disease compared to healthy patients, described the role of several cytokines (i.e., CC chemokines, CXC chemokines, proinflammatory cytokines, growth factors, proteolytic proteins, and cell adhesion cytokines) in the pathogenesis of the aneurysms. In this study, several cytokines (e.g., IL-1α, IL-1β, IL-2, and IL-17) and growth factors (e.g., insulin-like growth factor-1, transforming growth factor-α, and tumor necrosis factor-α) were reported to activate NGAL, a marker of neutrophil activation [36] that is involved in tissue injury [37,38]. In our previous study, we documented an increase in plasma levels and tissue expression of both MMP-9 and NGAL in patients with central and peripheral aneurysms [7]. In the present study, we confirmed the involvement of MMP-2, MMP-9, and NGAL in aneurysmal lesions (Figure 1), and we also demonstrated their plasma decrease during the follow-ups (Figure 2 and Figure 3). We also detected the protein expression levels of those biochemical mediators in aneurysmatic tissues, with no differences with respect to the location of the lesion. MMP-2 is derived from smooth muscle cells and fibroblasts and to a lesser extent macrophages [39], whereas MMP-9 is predominantly derived from macrophages and neutrophils [40]. Both are involved in the development of thoracic and aortic aneurysms [41,42]. On the other hand, NGAL levels reflect both inflammation and leukocyte activation in patients with arterial aneurysms and are correlated with aneurysm growth [43]. Of note, NGAL is released in the acute phase of inflammation by the secretory machinery of leukocytes and, in the glycosylated form, covalently binds to MMP-9 to make the MMP-9/NGAL complex. This complex, in turn, triggers an enhancement of the enzymatic activity of MMP-9 and protects MMP-9 from proteolytic degradation by TIMP1 and 2 proteins [37,44]. The crucial role of MMP-9 in the imbalance of the aortic wall drives the need to have pharmacological tools that can decrease its levels in the aneurysmal patient. In this view, we documented that several drugs/compounds are able to inhibit MMP expression [13,20]. Likewise, we reported that HMG-CoA reductase inhibitors (simvastatin and rosuvastatin) can block RAS prenylation, and thus are able to inhibit NF-κB signaling as well as decrease MMP-2 and MMP-9 protein expression in cell culture models [17,18]. In the present study, we reported that treatment with HMG-CoA reductase inhibitors at a high dose decreases the aneurysmal tissue levels of MMP-2, MMP-9, and NGAL (Figure 4). In an experimental study, Luan et al. [45] reported that statins inhibit in a dose-dependent manner the secretion of MMPs and decrease their collagenolytic, caseinolytic, and gelatinolytic activities. Moreover, other authors have documented that statins reduce the secretion of a broad spectrum of MMPs from macrophages and vascular endothelial cells, suggesting a beneficial effect of these drugs on these cells [46,47]. In our study, we documented that high potency statins compared to low potency ones have chemical characteristics that allow them to independently affect MMP-2, MMP-9, and NGAL in aneurysmal tissue levels, with no difference with respect to the site of the aneurysm. On the other hand, in the current study, we documented that 61% of patients in Group I and its relative subgroups, as well as 53.3% of patients in Group III, developed muscle pain as ADRs related to statin use. It is worth mentioning that the inhibition of HMG-CoA reductase blocks the synthesis of mevalonate, a precursor of farnesyl pyrophosphate that, in addition to being a substrate of cholesterol, is also the substrate for ubiquinone, also known as coenzyme Q synthesis [48]. Therefore, integrating coenzyme Q with supplements can be a strategy for reducing muscle pain mediated by statins [49,50]. Our study adds knowledge for clinical practice, but it also has some limitations. First, we measured the immune reactivity of MMP-2, MMP-9, and NGAL in the plasma of patients suffering from aneurysmal disease. These biochemical assays do not reflect the enzymatic activity of the protease. Second, the aneurysmal tissues were obtained from surgery patients in which the aneurysm had reached a specific size, so we do not know if the early stages of aneurysms have the same alterations described here. Third, more biochemical and mechanistic studies are required to determine the action of HMG-CoA reductase inhibitors, since NGAL decreased as well as MMP-9.

## 5. Conclusions

In conclusion, MMP-2, MMP-9, and NGAL levels seem to play a major role in the development of aneurysms, and their levels are modulated by statin treatment, suggesting that HMG-CoA reductase inhibitors have a role in the prevention of these clinical manifestations.

## Figures and Tables

**Table 1:** Enrolled patients in Group I (statin-treated), Group II (statin-free), and Group III (without aneurysm, taking statins, defined control group). Data are expressed as absolute values or as mean ± standard deviation.

Characteristics	Group I (n = 95)	Group II (n = 119)	Group III (n = 122)
Mean age (years)	71.5 ± 6.3	70.6 ± 6.4	69.3 ± 6.3
Male	65	78	68
Female	30	41	54
Aneurysm diameter	54.2 ± 15.2	49.3 ± 17.9	----
Cigarette smokers	42	63	59
Body mass index			
>30	12	10	22
25–29.9	70	94	83
18.5–24.9	13	15	17
<18.5	0	0	0
Glutamic oxaloacetic transaminase	33.3 ± 3.2	28.1 ± 4.9	26.7 ± 4.1
Glutamic pyruvic transaminase	32.5 ± 4.3	31.1 ± 3.6	29.3 ± 5.5
Platelet count	290.630 ± 21.315/µL	312.250 ± 28.530/µL	279.870 ± 53.210/µL
**Comorbidity**			
Blood hypertension	86	112	108
COPD	45	60	36
Hypercholesterolemia	95	----	68
Acute coronary syndrome	55	----	15
Diabetes type II	42	21	35
Diabetes type I	8	10	9
**Pharmacological therapy**			
Antihypertensive	86	112	108
Antiarrhythmic	32	----	2
Bronchodilators	45	60	36
Statins	95	----	68
Hypoglycemic drugs	39	16	35
Insulin	8	10	9
**ADRs related to statins**			
Myalgia	38		39
Gastrointestinal disturbance	12		16
Increase in transaminases	8		10
